# Death Rates from Malaria Epidemics, Burundi and Ethiopia

**DOI:** 10.3201/eid1301.060546

**Published:** 2007-01

**Authors:** Jean-Paul Guthmann, Maryline Bonnet, Laurence Ahoua, François Dantoine, Suna Balkan, Michel van Herp, Abiy Tamrat, Dominique Legros, Vincent Brown, Francesco Checchi

**Affiliations:** * Epicentre, Paris, France; † Médecins Sans Frontières, Paris, France; ‡ Médecins Sans Frontières, Brussels, Belgium; § Médecins Sans Frontières, Geneva, Switzerland; ¶ World Health Organization, Geneva, Switzerland; # London School of Hygiene, London, United Kingdom; Tropical Medicine, London, United Kingdom; 1 Current affiliation: Institut de Veille Sanitaire, Saint-Maurice, France

**Keywords:** Malaria, Plasmodium falciparum, epidemic, mortality, Burundi, Ethiopia, dispatch

## Abstract

Death rates exceeded emergency thresholds at 4 sites during epidemics of *Plasmodium falciparum* malaria in Burundi (2000–2001) and in Ethiopia (2003–2004). Deaths likely from malaria ranged from 1,000 to 8,900, depending on site, and accounted for 52% to 78% of total deaths. Earlier detection of malaria and better case management are needed.

*Plasmodium falciparum* malaria epidemics are poorly documented, partly because they occur in remote, underresourced areas where proper data collection is difficult. Although the public health problems from these epidemics are well recognized (*1*,*2*), quantitative evidence of their effect on death rates is scarce (*3*). Hospital-based death data, when available, provide a grossly incomplete picture because most malaria patients do not seek healthcare and, thus, these cases are not reported (*4*). Thus, current estimates (*2*) rely on extrapolations of limited site-specific or empirical observations. Accurate information is needed not only to improve our knowledge of malaria epidemics, but also to assess progress of malaria control initiatives that aim to decrease deaths from malaria worldwide by 50% by 2010 (*5*). We report community-based death rates from 2 *P. falciparum* malaria epidemics (Burundi, 2000–2001; Ethiopia, 2003–2004) in which Médecins Sans Frontières intervened.

Detailed information about these epidemics, their determinants, and their evolution is provided elsewhere (*6*). Briefly, the inhabitants of the Kayanza, Karuzi, and Ngozi provinces (population 1,415,900) of Burundi, which borders Rwanda, live in small farming villages, most at an altitude >1,500 m. Before the 2000–2001 epidemic, these areas were considered to have low malaria transmission. Rapid surveys of febrile outpatients confirmed the epidemic (>75% had *P. falciparum* infections; Médecins Sans Frontières, unpub. data). For all 3 provinces, 1,488,519 malaria cases were reported (attack rate 109.0%). Figure 1 shows the number of cases each month. In Kayanza, 462,454 cases were reported from September 2000 through May 2001 (attack rate 95.9%, average cases/month 51,383) (*7*); case counts peaked in January. In Karuzi, 625,751 cases were reported from October 2000 through March 2001 (attack rate 202.8%, average cases/month 10,429); case counts peaked in December (*7*). Ngozi reported 400,314 malaria cases from October 2000 through April 2001 (attack rate 67.7%, average cases/month 57,187); case count peaked in November (*7*).

**Figure 1:**
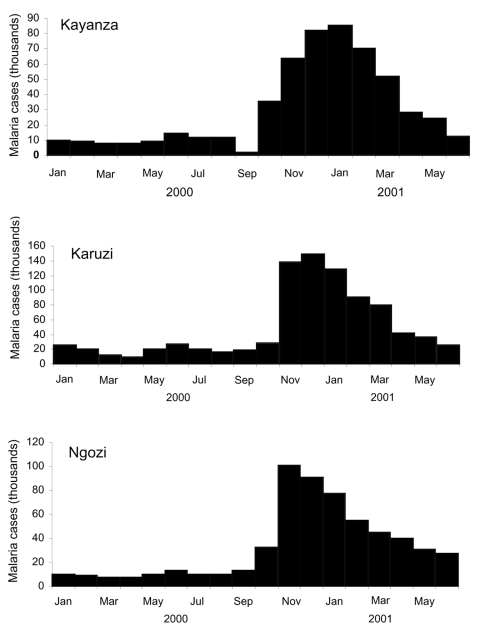
Clinical cases of malaria reported from Kayanza, Karuzi, and Ngozi provinces (Burundi), January 2000–June 2001.

Damot Gale district (286,600 inhabitants, altitude 1,600–2,100 m), considered a low-transmission area, is located in Wolayita Zone, Southern Nations Nationalities and Peoples Region, central Ethiopia**.** The malaria epidemic was confirmed locally by a sharp increase in *P. falciparum*–positive results among children treated in Médecins Sans Frontières feeding centers; the increase started in July 2003 (*6*). Reported caseload decreased in August and September, probably because of drug shortages and subsequent untreated and unreported patients; caseload rose sharply in October, November, and December (Figure 2). During these 3 months in 2003, 10,308 cases were reported by the 8 district health facilities (attack rate 3.6%, average no. cases/month 3,436), more than 10-fold the corresponding total in 2002 (n = 744) (Médecins Sans Frontières, unpub. data).

**Figure 2:**
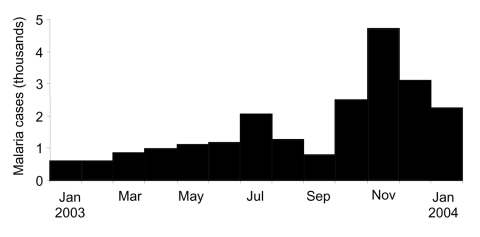
Clinical cases of malaria reported from Damot Gale health facilities (Ethiopia), January 2003–January 2004.

## The Study

During the epidemics, a retrospective survey of deaths was conducted at each site. Surveys were approved by local authorities, and respondents gave oral consent. Thirty clusters of 30 households were selected by using 2- or 3-stage sampling (*8*). Households were defined as groups of persons who slept under the same roof under 1 family head at the time of the survey; occasional visitors were excluded. Selection within each cluster followed a standard rule of proximity (*9*). Information collected included number, age, and sex of persons living in the household; number of deaths (age, sex, and date of death) since the beginning of the recall period; and cause of death. Malaria was defined as the probable cause if a decedent’s household reported “presence of fever” (Burundi) or “fever and shivering without severe diarrhea or severe respiratory infection” (Ethiopia). Recall periods were defined by easily recognizable starting dates (Table 1).

**Table 1:** Results of retrospective surveys of deaths in Burundi (2000–2001) and Ethiopia (2003–2004)*

	Burundi			Ethiopia
	Kayanza	Ngozi	Karuzi	Damot Gale
Dates of survey	December 2000	February 2001	March 2001	January 2004
Beginning of recall period	Anniversary of Prince Rwagasore murder†	4 wk before anniversary of Prince Rwagasore murder	Anniversary of Prince Rwagasore murder	Last Meskal‡
Recall period, d	53	155	153	125
No. persons in sample	4,308	3,639	4,925	5,619
No. children <5 y in sample (%)	729 (16.9)	639 (17.5)	961 (19.5)	1,167 (20.8)
No. deaths during recall period	23	100	87	148
No. deaths, children <5 y	14	51	45	51
CMR (95% CI)	1.0 (0.6–1.7)	1.8 (1.3–2.3)	1.1 (0.9–1.5)	2.1 (1.5–2.9)
U5MR (95% CI)	3.6 (1.7 - 7.2)	5.0 (3.3–7.4)	3.0 (2.0–4.4)	3.4 (2.3–5.1)
No. deaths probably due to malaria (%)	18 (78.3)	58 (58.0)	45 (51.7)	106 (66.0)
No. deaths probably due to malaria, children <5 y (%)	9 (64.3)	27 (53.0)	24 (53.3)	42 (82.3)
Probable malaria-specific mortality rate (95% CI)	0.8 (0.4–1.5)	1.0 (0.7–1.5)	0.6 (0.4–0.8)	1.5 (1.0–2.1)
Probable malaria-specific mortality rate, children <5 y (95% CI)	2.3 (0.9–5.4)	2.6 (1.5–4.6)	1.6 (0.9–2.8)	2.8 (1.9–4.1)

*CMR, crude mortality rate (deaths/10,000/d); CI, confidence interval; U5MR, mortality rate for children <5 y of age (deaths/10,000/d). †October 13, 2000. ‡September 28, 2003.

Data were analyzed by using EpiInfo (Centers for Disease Control and Prevention, Atlanta, GA, USA). Death rates were expressed as deaths/10,000 persons/day, and 95% confidence intervals (CIs) were adjusted for design effects. Mortality rates were compared with standard emergency thresholds of 1 death/10,000/day (crude mortality rate [CMR]) and 2 deaths/10,000/day (under 5 mortality rate [U5MR]) (*10*). Excess number of deaths probably due to malaria was estimated by applying the specific death rates due to self-reported malaria to the population and time period covered by each survey.

CMR and U5MR exceeded respective emergency thresholds (Table 1). In the total population, proportion of deaths probably due to malaria varied from 51.7% (Karuzi) to 78.3% (Kayanza) and from 53.0% (Ngozi) to 64.3% (Kayanza) for children <5 years of age (Table 1).

Deaths probably due to malaria ranged from 1,000 in Kayanza to 8,900 in Ngozi; >50% were among children <5 years (Table 2). Estimates reflect only portions of the epidemic periods (Table 2). When surveys covered most of the epidemic duration (74% in Ngozi, 85% in Karuzi, 83% in Damot Gale), malaria was the probable cause of death for a comparable proportion of the population (1.5% [8,900/574,400] in Ngozi, 0.9% [2,800/308,400] in Karuzi, and 1.9% [5,400/286,600] in Damot Gale).

**Table 2:** Estimated number of deaths, total population and children <5 years of age, Burundi (2000–2001) and Ethiopia (2003–2004) malaria epidemics*

	Burundi			Ethiopia
	Kayanza	Ngozi	Karuzi	Damot Gale
Estimated source population (total)	246,500	574,400	308,400	286,600
Estimated no. children <5 y†	41,900 (17%)	103,400 (18%)	61,100 (19.8%)	59,900 (20.9%)
Recall period, d (approximate proportion of entire epidemic period)	53 (20%)	155 (74%)	153 (85%)	125 (83%)
Estimated no. deaths, all causes (95% CI)	1,300 (800–2200)	16,000 (11,600–20,500)	5,200 (4,700–7,100)	7,500 (5,400–10,400)
Estimated no. deaths, all causes, children < 5 y (95% CI)	800 (400–1,600)	8,000 (5,300–11,900)	2,800 (1,900–4,100)	2,500 1,700–3,800)
Estimated no. deaths probably due to malaria (95% CI)	1,000 (500–,2,000)	8,900 (6,200–13,400)	2,800 (1,900–3,800)	5,400 (3,600–7,500)
Estimated no. deaths probably due to malaria, children <5 y (95% CI)	500 (200–1,200)	4,200 (2,400–7,400)	1,500 (800–2,600)	2,100 (1,400–3,100)

*CI, confidence interval.  †Using estimates from respective surveys, the mid-period proportion of children <5 years was calculated as follows: (children alive at end of period + [0.5 × deaths for children <5 y during period])/(all persons alive at end of period + [0.5 × all deaths during period]).

## Conclusions

We provide novel data based on representative population sampling, rather than health facility–based reporting. *P. falciparum* epidemics seem responsible for high death rates: the estimated number of deaths probably due to malaria at our sites (≈18,000) represents about 10% of the worldwide total estimated annual deaths due to epidemic malaria (*2*).

The limitations of retrospective mortality surveys are well known (*11*); hence, results should be interpreted with caution. Reporting bias was minimized by defining a limited recall period and by training interviewers extensively. In Kayanza, the survey was conducted before the epidemic peak; the estimated death rate may have been lower than average for the entire epidemic, which may have led to underestimation of the true death rate. Generally, postmortem diagnosis of malaria at the household level is difficult, and even advanced verbal autopsy techniques (not used in these surveys due to lack of skilled human resources) are of limited accuracy (*12*). Decedents’ next of kin may underreport or overreport certain signs and symptoms. Malaria deaths may thus have been overestimated, particularly in Burundi, where fever was the sole criterion of probable malaria; use of this 1 criterion may have masked other causes, such as acute respiratory infection. Furthermore, in 3 of the areas surveyed (Kayanza excepted), the epidemics occurred concurrently with nutritional crises. Malnutrition as a cause of death could not be assessed because of its implication in various infectious diseases, but high prevalence of malnutrition is usually associated with excess U5MR (*13*). Nevertheless, mortality rates among persons ≥5 years of age (CMR – [U5MR × proportion of children <5 years in survey sample]/[1 – proportion of children <5 years in survey sample]) were also elevated. Rates ranged from 0.5 in Kayanza to 1.7 in Damot Gale, higher than the expected rate of 0.27 in sub-Saharan Africa (*14*). In the absence of other specific causes of acute death for adults, we speculate that malaria was largely responsible for these excess deaths.

At all sites, early warning systems were not operational and surveillance was ineffective, which led to substantial delays in epidemic detection (*6*). First-line treatment regimens (chloroquine in Burundi, sulfadoxine/pyrimethamine in Ethiopia) were not very effective. In Damot Gale, access to treatment was poor (data not shown), probably due to the dearth of health facilities. All these factors may have exacerbated the epidemics and contributed to excessive death rates.

Early diagnosis and prompt treatment of malaria remain cornerstones of the global malaria control strategy (*15*). The degree to which these interventions will be made available will largely determine the death rates in future epidemics.
